# Is transport distance correlated with animal welfare and carcass quality of reindeer (*Rangifer tarandus tarandus*)?

**DOI:** 10.1186/s13028-017-0286-z

**Published:** 2017-03-15

**Authors:** Sauli Laaksonen, Pikka Jokelainen, Jyrki Pusenius, Antti Oksanen

**Affiliations:** 1 Department of Veterinary Biosciences, Faculty of Veterinary Medicine, University of Helsinki, P.O. Box 66, 00014 Helsinki, Finland; 2 Estonian University of Life Sciences, Kreutzwaldi 62, 51014 Tartu, Estonia; 3 Faculty of Veterinary Medicine, University of Helsinki, P.O. Box 66, 00014 Helsinki, Finland; 4 Statens Serum Institut, Artillerivej 5, 2300, Copenhagen S, Denmark; 5 Natural Resources Institute Finland, Yliopistokatu 6, 80100 Joensuu, Finland; 6 Production Animal and Wildlife Health Research Unit, Finnish Food Safety Authority Evira, Elektroniikkatie 3, 90590 Oulu, Finland

**Keywords:** Reindeer, Meat inspection, Stress, Handling, Transport, Trauma, Aspiration of rumen content, Abnormal odour, Welfare

## Abstract

**Background:**

Slaughter reindeer are exposed to stress caused by gathering, handling, loading and unloading, and by conditions in vehicles during transport. These stress factors can lead to compromised welfare and trauma such as bruises or fractures, aspiration of rumen content, and abnormal odour in carcasses, and causing condemnations in meat inspection and lower meat quality. We investigated the statistical association of slaughter transport distance with these indices using meat inspection data from years 2004–2016, including inspection of 669,738 reindeer originating from Finnish reindeer herding areas.

**Results:**

Increased stress and decreased welfare of reindeer, as indicated by higher incidence of carcass condemnation due to bruises or fractures, aspiration of rumen content, or abnormal odour, were positively associated with systems involving shorter transport distances to abattoirs. Significant differences in incidence of condemnations were also detected between abattoirs and reindeer herding cooperatives.

**Conclusions:**

This study indicates that in particular the short-distance transports of reindeer merit more attention. While the results suggest that factors associated with long distance transport, such as driver education, truck design, veterinary supervision, and specialist equipment, may be favourable to reducing pre-slaughter stress in reindeer when compared with short distance transport systems, which occur in a variety of vehicle types and may be done by untrained handlers. Further work is required to elucidate the causal factors to the current results.

**Electronic supplementary material:**

The online version of this article (doi:10.1186/s13028-017-0286-z) contains supplementary material, which is available to authorized users.

## Background

In Fennoscandia, semi-domesticated reindeer are grazing most of the year freely on natural pastures. Traditionally, reindeer were slaughtered on the field during round-ups, and no transportation to slaughter houses was needed. Field slaughter facilities were erected ad hoc until the 1980s, at which time slaughtering moved to more developed and modern export abattoirs. Nowadays, most of the reindeer in Finland are slaughtered in officially approved abattoirs. The development of a network of regional abattoirs created the need for intensive transportation of live reindeer and led to the evolution of reindeer transportation [1].

Most of the transportation of live reindeer by motor vehicles takes place for slaughter in autumn and early winter [1]. The vehicles include vans and trailers, and for longer distances, special reindeer transport trucks. In addition to transporting to slaughter, vehicle transportation is occasionally used when reindeer are moved between pastures, or to supplementary feeding sites or corrals for winter months. Motor vehicles, helicopters, snow mobiles and quad bikes (ATVs) are used as aids when herding and gathering reindeer for summer or autumn (slaughter) round-ups.

In Finland, all animal transportation is regulated by the Finnish Animal Transport Act (1429/2006) and the Council Regulation (EC) No 1/2005 on the protection of animals during transport and related operations. There is no specific act for reindeer and thus these regulations are also valid for the transportation of reindeer.

Any external stimulus that challenges homeostasis can be viewed as a stressor to animals [2]. Emotional stimuli are the most common and important stressors in animals with a highly developed nervous system [3–6]. Animals react differently when they are captured, restrained, or immobilized. Even animals that seem to adapt to the situation may suffer from stress and be vulnerable to related damaging changes [7, 8]. Transportation is known to cause substantial stress in domesticated animals [9, 10] and it likely provokes an even more severe stress response in semi-domesticated animals.

The transportation and its impact on the welfare of semi-domesticated reindeer has been an issue of concern in Fennoscandia. The transportation to slaughter includes pre-slaughter handling of the animals; rounding up, herding, holding in enclosures, manual handling, loading, road transport, and unloading. After the transport of reindeer, traumas are commonly found [11–13]. Trauma may be caused by a physical impact by antlers, hooves, metal or wooden projections, or animals falling and being trampled on by others. Such trauma can take place any time during handling, transport, holding, or stunning. Bruises can vary in size, and be superficial or severe and may be seen in different parts of the carcass or even over the entire carcass.

Reindeer, like wild animals, are susceptible to stress caused by human presence, handling, capturing, and transportation. Manual handling and restraint have been found to be one of the major stress factors for reindeer [12–15]. There are indications of a cumulative effect of repeated stress events. In herded reindeer, stress associated lesions, such as abomasal haemorrhage, as well as myocardial and muscular degeneration have been described [11, 13–15]. Physical trauma also cause stress, but as they are often the result of aggression by other animals in the crate, they may also be considered a result of stress behaviour [12].

The detrimental effects of pre-slaughter handling on blood chemistry (aspartate transaminase (ASAT), urea, cortisol) and muscle glycogen stores can cause increased pH values resulting in lower meat quality; these effects have been demonstrated in several Fennoscandian studies [12–22]. Management and handling stress are also reflected by an increase in numbers of both immature and mature neutrophils and a decrease in lymphocyte count, which is correlated to the degree of stress to which the animals have been exposed. Prolonged exposure to stress results in a decrease in the number of eosinophils in peripheral blood [12].

Rehbinder et al. [12], Rehbinder [13] and Wiklund et al. and [14, 20] demonstrated that the use of a lasso to capture reindeer for slaughter was the most stressful handling procedure among those studied. In these studies, lorry transport, helicopter herding, and fixation of animals by hand without the use of a lasso resulted in lower stress responses as measured by meat pH values, blood metabolites used as stress markers i.e. ASAT, urea and cortisol, and abomasal lesions, compared to the lasso capture procedure. The calves have been reported to have higher muscle pH [19] and plasma urea [12, 13] values after herding and handling stress than adults. The authors concluded that calves are more susceptible to stress than adult animals due to more vigorous physical exertion depleting their energy stores more rapidly. Exhausted animals were in general found to have extremely high meat pH values, with 31.1% of the carcasses being classified as intermediate Dark, Firm and Dry meat (DFD) (5.8 < pH < 6.2) and 31.2% as DFD (pH > 6.2) [19].

Natural long-duration stress, such as harsh weather and snow conditions especially in winter time, can also cause poorer meat quality; glycogen stores are used before slaughter and the pH-value remains high [14, 23]. In addition, the animals’ physical condition and nutritional status have a considerable effect on their ability to tolerate various stress factors, such as lorry transport and holding [20].

Hyvärinen et al. [16] found elevated serum urea values associated with reindeer gatherings. Furthermore, the levels were correlated with the distance of the drive and time spent in the corral. The study by Wiklund et al. [15] confirmed the finding that a “stress-flavour” could develop in reindeer meat after intensive pre-slaughter handling of the animals. The animals captured by use of lasso or herded by helicopter for prolonged three days had the highest scores of an unpleasant, strong, even acrid smell, which was described by a trained expert panel as a pungent odour, sickeningly sweet odour, sharp flavour, and sickeningly sweet flavour [15]. It is common knowledge among reindeer herders that animals that have been exposed to stressful pre-slaughter handling give meat with an unfavourable odour [15] which is sometimes referred to as “urine smell” [1, 13]. Several studies have tried to correlate the concentrations of substances such as putrescine, spermidine, spermine, creatine, creatinine, and dimethylamine in reindeer meat and plasma with the presence of ‘stress-flavour’ in the meat, but the issue is still unresolved, as reviewed by Wiklund [15]. According to Rehbinder [13], depletion of muscular glycogen stores, increased catabolism of muscular protein, muscular degeneration, and increased blood-urea levels cannot be excluded as a cause of an altered and bad taste of the meat.

In Sweden, lorry transport did not affect the ultimate pH of the muscles of bulls and calves and the incidence of high pH and intermediate DFD in reindeer hinds was greater only after transport of more than 500 km [19]. In Finland, Nieminen et al. studied the impact of transportation of reindeer in 1993 [24] and in 2000 [25]. In these studies, the transportation distances varied from 30 to 400 km and transportation times from 1 to 5.5 h. The reindeer were reported to be peaceful and in good shape after transportation and only minor bruises were detected.

Aspiration of rumen content during stunning is a common finding during slaughter in production ruminants, leading to the condemnation of lungs at meat inspection [26]. There is, however, apparently, no published data of the causes or incidence of this phenomenon. Aspiration of rumen content is also often seen during reindeer meat inspection. Hanssen et al. [27] reported the transportation of reindeer on lorries to result in more liquid rumen content. In addition, a marked stress response with abomasal erosions or ulcers will affect the digestive tract and its utilization of fodder [13, 15]. It is also common that digestive disorders occur amongst reindeer after supplementary feeding [1, 28]. Reindeer are usually supplementary fed with silage and pellets [1], which often leads to fullness and distension of the rumen. These feeds are medium or high protein rich, which greatly increases their water requirements. For example, adult female reindeer eating pellets have been reported to drink 3.2–3.5 l of water per day, while reindeer fed lichens drank only 0.1 l per day [29]. More liquid rumen content may perhaps predispose to regurgitation and aspiration of rumen content during stunning.

There has been a lot of public debate concerning the long-distance transportation of reindeer by motor vehicles; in particular, with regards to the effect of transportation on the wellbeing of the reindeer. The aim of this study was to partially respond to these concerns by exploring whether the distance of the transportation of live reindeer to abattoirs is associated with higher rates of meat condemnations. We focused on injuries, bruises and fractures, but stress-related abnormal odour and aspiration of rumen content were also surveyed. The outcomes investigated are not only indicators of compromised welfare but also relevant for the brand and reputation of reindeer meat production.

## Methods

The reindeer population data were obtained from database of the Reindeer Herders’ Association. The reindeer meat inspection data were collected from official documents from meat inspection veterinarians (see Additional file 1) at all the approved reindeer abattoirs of Finland, from the slaughter seasons (autumn and early winter) 2004–2005 to 2015–2016. The meat inspection and hygiene control in abattoirs was conducted by veterinarians who work under the control of Regional State Administrative Agencies of Lapland. For this study, the number of bruises and fractures and stress-related abnormal odour and aspiration of rumen content, which lead to partly or total carcass condemnations for human consumption, were included. We recorded the number of inspected reindeer originating from Finnish reindeer herding areas (669,738 reindeer from 4208 slaughter batches), the number and the percentage of condemnations due to bruises or fractures, aspiration of rumen content during stunning, and organoleptic evaluations of abnormal odour.

### Statistical analyses

**Fig. 1:**
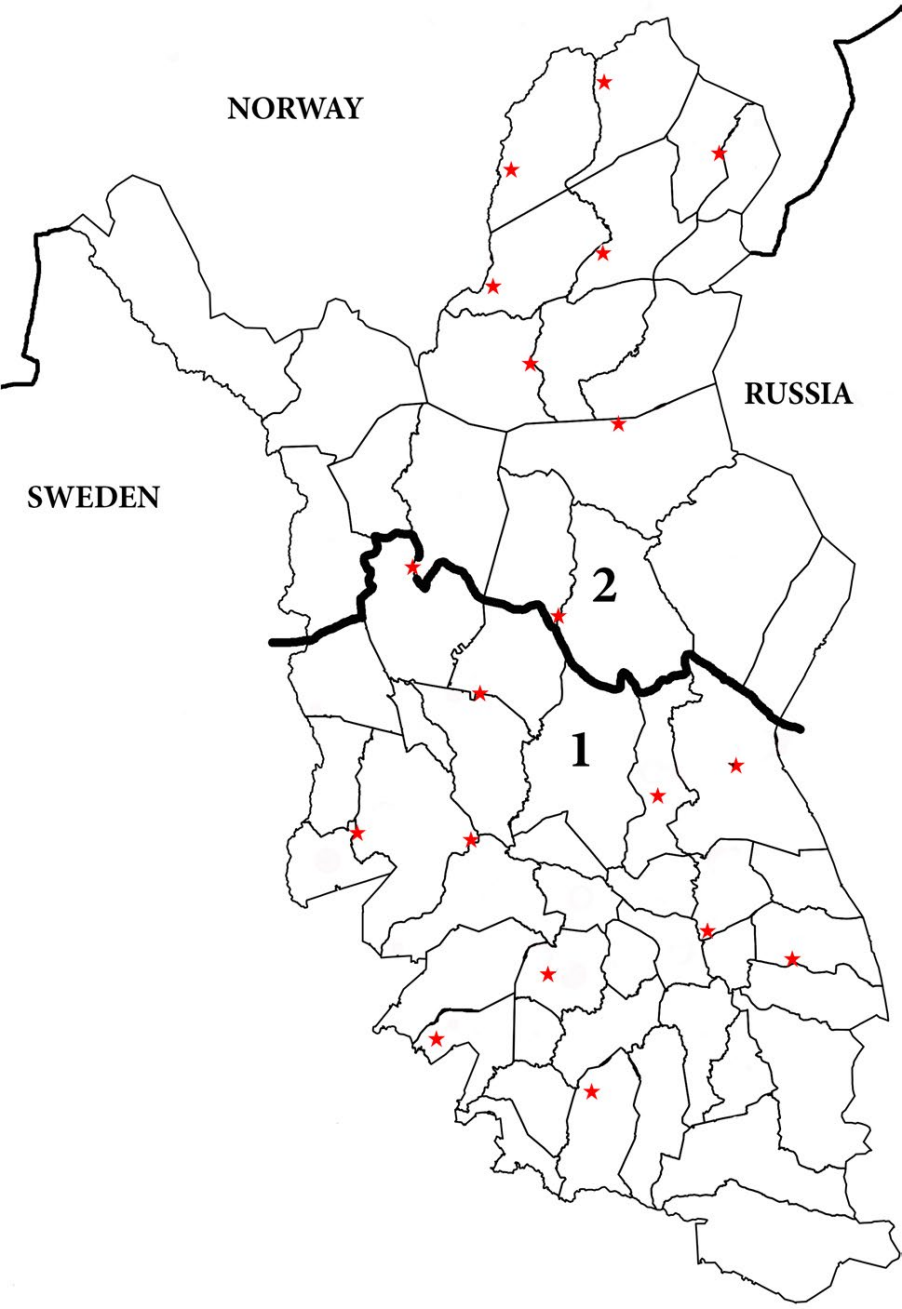
Finnish reindeer herding area indicating the area specifically intended for reindeer herding (Area 2, northern part) and the remainder (Area 1, southern part). *Fine lines* are the borders of reindeer herding cooperatives and the *red stars* are official reindeer abattoirs

For statistical analyses, the transportation distances were classified into five categories: 0 = 0 km, reindeer walk from the round-up corral to abattoir; (1) 1–60 km; (2) 61–150 km; (3) 151–300 km; (4) >300 km. The transport distances for each slaughter batch were defined to be the shortest distance along the road from the geographical centre of the reindeer herding cooperative to the abattoir. In addition, the reindeer were classified according to their region of origin: The Finnish reindeer herding area was divided into the area specifically intended for reindeer herding (Area 2, northern part) (Finnish Reindeer Husbandry Act, 14.9.1990/848) and the remainder (Area 1, southern part) (Fig. 1).

We analysed the association between the occurrence of bruises or fractures, abnormal odour, and aspiration of rumen content leading to condemnations and the transport distance of reindeer to abattoirs using Spearman’s rank correlation coefficient. The relationship between the regional origin of reindeer and the proportion of condemnations was analysed using two by two contingency tables and Chi squared tests.

The comparison of proportion of condemnations between abattoirs and 56 reindeer herding cooperatives was made by using non-parametric Kruskal–Wallis test using slaughter batch as a unit of observation. In one abattoir, animals were bled in the horizontal position (as opposed to vertical position). To determine if the proportion of animals aspirating rumen contents differed in this abattoir compared to the others, we used a post hoc multiple comparison analysis identifying homogenous subsets (significance level 0.05).

All statistical analyses were conducted using SPSS 19 software.

## Results

The reindeer population in Finland during the study period was on average 197,807 (190,776–209,365) individuals, of which an average of 102,778 (71,580–124,152) were slaughtered annually. Of the slaughtered reindeer, 77% were calves (6–8 months old). Approximately 74% of the reindeer were slaughtered in 19 EU-approved reindeer abattoirs; the rest were slaughtered in the field for private consumption and direct marketing (Regional State Administrative Agencies of Lapland).

The inspection data of 669,738 reindeer (Area 1, 234,821; Area 2, 434,917) from 4208 slaughter batches (Area 1, 2318; Area 2, 1890) were included in the study; data from 333 slaughter batches were excluded because of missing information. Eighty-three percent of the reindeer were slaughtered during October–December. The average distance that animals were transported to slaughter was 87 km (range: 0–450 km) in the whole area, 62 km (10–390 km) in the southern area (Area 1), and 117 km (0–450 km) in the northern area (Area 2).

The meat inspection findings associated with part or whole carcass condemnation from 2004 to 2016 are presented by region in Table 1, (Fig. 1). Bruises and fractures, and aspiration of rumen content, were more common in Area 1 compared to Area 2 (Table 1; χ^2^ = 50.58, df = 1, P < 0.001; χ^2^ = 2212.93, df = 1, P < 0.001; respectively).

**Table 1:** Indicators of compromised welfare of reindeer leading to meat inspection condemnations in Finland (2004–2016)

Origin of reindeer^a^	No of reindeer	Mean transportation distance (km)	Bruises and fractures (n, %)	Aspiration of rumen content (n, %)	Abnormal odour (n, %)
Area 1	234,821	62	1533 0.6%	1265 0.54%	65 0.03%
Area 2	434,917	117	2246 0.5%	36 0.008%	126 0.03%
Whole area	669,738	87	3779 0.6%	1301 0.2%	191 0.03%

^a^The map showing the areas: Fig. 1

The transport distance to the abattoir was negatively correlated with the number of condemnations due to bruises/fractures (Spearman’s rank correlation r_s_ = −0.20, N = 4208, P < 0.0005), aspiration of rumen content (r_s_ = −0.04, N = 4208, P = 0.005), and abnormal odour (r_s_ = −0.04, N = 4208, P = 0.011). The correlations of aspiration of rumen content and abnormal odour with travel distance were however weak, whereas the negative correlation between the condemnations due to bruises and fractures and transport distance indicated a definite and strong relationship.

The number of bruises and fractures leading to condemnations during reindeer meat inspections are presented by the five transport distance categories in Table 2. Multiple comparison after Kruskal–Wallis ANOVA indicated that the incidence of bruises and fractures was lower in the transportation distance category 0 (no transport) than in the transportation distance category 1 (1–60 km) (P < 0.05). The slaughter batches were smaller in the categories of short transport than in the category of no vehicle transport (280 and 123 reindeer respectively).

**Table 2:** Bruises and fractures leading to condemnations during reindeer meat inspections, by transport distance categories

Transport distance		Inspected reindeer (n)	Inspections (n)	Reindeer in inspections (mean)	Bruises and fractures (n)	Bruises and fractures (%)
km	Category					
0	0	129,521	464	280	590	0.45
1–60	1	260,688	2123	123	1970	0.75
61–150	2	124,402	1038	120	743	0.60
151–300	3	89,124	328	272	386	0.43
≥301	4	66,005	255	259	90	0.13
Total		669,740	4208	159	3779	0.56

There were significant differences between abattoirs in the incidence of bruises and fractures (range 0.20–2.02%, mean 0.71%, SD 2.93%; Kruskal–Wallis test, H = 564.73, df = 19, P < 0.0005), rumen content aspirations (range 0.00–2.47, 0.50%, SD 3.02%; Kruskal–Wallis test, H = 660.94, df = 19, P < 0.0005) and abnormal odour (range 0.00–0.74%, 0.08%, SD 1.77%; Kruskal–Wallis test, H = 109.54, df = 19, P < 0.0005) as well as between all 56 reindeer herding cooperatives (range 0.00–7.69%, 0.74%, SD 3.07%; Kruskal–Wallis test, H = 523.07, df = 55, P < 0.0005), (range 0.00–3.22%, 0.53%, SD 3.15%; Kruskal–Wallis test, H = 612.35, df = 55, P < 0.0005), (range 0.00–0.68%, 0.08%, SD 1.85%; Kruskal–Wallis test, H = 91.81, df = 55, P = 0.001), respectively.

Multiple comparisons identifying homogenous subsets (significance level 0.05) indicated that condemnation caused by aspiration of rumen content was on average higher in the abattoir in which the bleeding after stunning was done on animals that were lying horizontally (2.4%), compared to those in which the animals were bled while hanging vertically (0.2%).

## Discussion

Our results indicate that the studied indicators of compromised physical welfare of slaughter reindeer are associated with the distance of transportation for slaughter: short transport distances were associated with more compromised reindeer welfare and carcass quality. In addition, we found that the incidence of these indicators varied significantly among reindeer herding cooperatives and abattoirs.

Bruises and factures were negatively associated with transport distance. This perhaps unexpected finding could be explained by the fact that most long-distance transport of slaughter reindeer is done with trucks that are specifically designed for the transportation of reindeer. These trucks must be inspected and approved by authorities and the driver must be specifically educated to be qualified and licenced for long-range animal transportation. The trucks have specially designed ramps for loading and unloading reindeer with minimal manual handling and they are sectioned into several pens that allow separation of calves from adult animals, which markedly reduces bruising and other injuries [11]. Conversely, transportation of reindeer for short (under 65 km) distances does not require any qualifications or approvals for the drivers nor the vehicles. Vans or trailers, which may in some cases be unfit, are used for the short-distance transportation, and the loading and unloading is done manually, reindeer by reindeer. Our results, therefore, suggest that the quality of the method of transportation more than compensates the potential negative effects of long-distance transportation. However, the finding that lower proportion of reindeer that were slaughtered without vehicle transport had traumatic lesions than reindeer that underwent short transport, indicates the impact of manual loading, unloading and the suitability of the transport vehicle on the injuries.

Our result that the proportion of reindeer having bruises/fractures detectable in meat inspection did not increase with the distance of transportation to abattoir is in accordance with former studies on the effects of transportation. In line with our conclusions, previous studies [19, 20, 24, 25], reported that lorry transport did not significantly impair reindeer meat quality indices. In addition, the historical meat inspection data from years 1980–1986 [33] indicated that bruises and fractures were common and were detected in 5.3% of the reindeer. This was in spite of the fact that only 11.5% of the reindeer were transported to the approved regional abattoirs and the rest were slaughtered in the field in connection to, or very near the round-up places, practically with no need of vehicle transport [33].

The significant differences in the appearance of bruises and fractures between individual cooperatives and abattoirs may be due to the different handling practises. For example, round-ups and their timing, maintenance of enclosures, transport corridors and ramps, routines for loading and unloading the animals, waiting times in corrals and in abattoirs, and understanding the natural behaviour, as highlighted in literature [12–15, 34], as well as the degree of tameness of reindeer [13], may differ. There are no stunning pens in Finnish reindeer abattoirs to support the animal, but the restraint is done manually, so bruising is also possible during the stunning and could occur also after stunning when the animal collapses on the floor [35].

In total, the current slaughter welfare conditions in Finland can be considered relatively good, with 0.6% of slaughter reindeer having detectable injuries, in particular when comparing with the situation in period 1980–1986, when bruises and fractures were reported in 5.6% of reindeer [33]. The situation has evolved in the same way also in Sweden: during 2000–2007, the proportion of injuries was 1% [36] and during 2006–2013, trauma or fractures were registered in 0.13% of reindeer carcasses and in 0.88% of the heads [37]. It must be noted that our data do not include the traumas in the head, which are typically seen in antlers, because such trauma do not usually lead to condemnations.

The apparently reduced incidence of bruises and fractures is likely due to the increased information and education delivered by the Reindeer Herders’ Association and related organizations and authorities which has led to improved handling methods and transports. One contributing factor can also be the fact that in Finland, especially in the northern reindeer herding area, veterinarians are present practically in every bigger slaughter round-up, administrating anti-parasitic treatment and at the same time doing animal welfare work, for example inspecting the fitness of animals for transportation.

Rumen content can become more liquid because of handling stress [13, 15], transportation [27] and supplementary feeding [28, 29]. This may predispose animals to aspiration of liquid rumen contents during or after stunning. In our study, aspiration of rumen content was reported in 0.2% of Finnish slaughter reindeer. The negative correlation to the distance of slaughter transport was not as clear as in bruises and fractures, while there were significant differences between individual cooperatives and abattoirs. In addition, aspiration of rumen content was a significantly more common finding in the reindeer from Area 1 compared to Area 2, reflecting decreased welfare and supporting that it could be associated with handling, transport, behavioural and feeding stress. In the southern area, reindeer are often held in lairage, waiting for the herd to grow big enough for the slaughter batch, sometimes several days. During that time, they are supplementary fed, usually with silage and pellets, which often leads to the fullness and distension of the rumen (unpublished observations).

During stunning, especially if the bolting accuracy or cartridge strength are not ideal, the unconsciousness is not immediate and reindeer continue to breathe which can result in regurgitation and aspiration of rumen content. It is known that slaughter without stunning (ritual slaughter) increases the possibility of aspiration of blood and, in the case of ruminants, rumen content [30]. All Finnish reindeer abattoirs use penetrating captive bolt pistols to stun reindeer. It is concluded that an animal effectively stunned with a penetrating captive bolt pistol, as indicated by the presence of certain signs and the absence of others, like failure to collapse, rhythmic breathing, eyeball rotation and, positive corneal reflex, has little possibility of a reversal of the brain function [31]. However, in cattle, the prevalence of shallow depth of concussion following captive bolt shooting was 8% for all cattle and 15% for young bulls, and 2.7% of animals maintained spontaneous breathing [32]. It is likely that this happens also during reindeer slaughter.

The finding that aspiration of rumen content was more common if the bleeding was done when the reindeer were in horizontal position compared to those abattoirs where reindeer were in a hanging position, was not surprising. It is logical that the regurgitation can reach lungs more easily if the animal is in horizontal position during commonly occurring reflexive gasping [32]. Aspiration of rumen content is also a concern for meat hygiene, since potential pathogens are transported to the clean side of the abattoir and can contaminate other carcasses and organs [38, 39].

“Stress flavour” occurs in reindeer meat after intensive handling of the animals prior to slaughter [15, 18, 20]. In our study, the finding registered was abnormal odour, which may include also other odours diagnosed by meat inspectors. A strong abnormal odour which lead to condemnations was diagnosed in 0.03% of reindeer. The real incidence of abnormal flavour is likely much higher, for example, Hanssen and Skei [40] detected moderate ammonia-like taint in two and a weak taint in seven samples of 29 reindeer which had been transported in lorries before slaughter. Nevertheless, our results show that the same trend as for bruises, fractures and rumen content aspiration was also detected in abnormal odour, having a negative correlation with transport distance and with significant differences between abattoirs and cooperatives. These patterns are in accordance with the published views that detecting abnormal odour is related to stress [15, 18, 20, 27, 40].

There are many more factors associated with transport and connected operations that could contribute to the observed effect of transport distance and that were not covered in this work. These include handling, holding, habituation, sex effect, socialisation, stunning, and the slaughter process. The differences in incidence between abattoirs and herding cooperatives reinforce this further.

Our study was based on the observations and meat inspection findings made by several veterinarians, which may cause bias on the results. All the reindeer meat inspectors have participated in education for meat inspection and harmonizing meat inspection decisions, and the reporting is simple. The strengths of the study include a long study period (>10 years) and a large number of observations, and the data were collected from official meat inspection decisions of condemnations. Because minor lesions and deep bruises, are not always detectable in the meat inspection, the true incidence of these indicators is likely higher.

## Conclusions

Long distance transport of reindeer in approved reindeer trucks and conducted by educated drivers was associated with less stress and trauma to the animals than transport for short distances, the latter requiring more manual handling, being conducted in a variety of vehicle types, and by untrained handlers. Although the welfare of reindeer during transportations and connected activities has improved, significant differences in incidence of bruises or fractures, aspiration of rumen content, and abnormal odour were detected between abattoirs and between reindeer herding cooperatives, emphasizing that there still is room for improvement. Although further research is required to elucidate the impact of all factors that are involved in transportation and that may be associated with stress and welfare of reindeer, the results of this study indicate that in particular the short-distance transports and related operations merit more attention. This is likely not limited to slaughter transport, but rather relevant to all transportation of reindeer e.g. to a feeding corral or to a different pasture area.

## Additional file

**Additional file 1.** Meat inspection decision for reindeer slaughter in Finland (translated from Finnish).
