# Supplementary material for: Is transport distance correlated with animal welfare and carcass quality of reindeer (Rangifer tarandus tarandus)?
Source: Acta Vet Scand. 2017 Mar 15;59:17. doi: 10.1186/s13028-017-0286-z (PMC5353846; doi:10.1186/s13028-017-0286-z)
Supplement: Supplementary file 1 — Additional file 1. Meat inspection decision for reindeer slaughter in Finland (translated from Finnish). [file 13028_2017_286_MOESM1_ESM.pdf]

Aluehallintovirasto
Guovlohálddahusdoaimmahat
Lappi

REGIONAL STATE ADMINISTRATIVE AGENCIES OF LAPLAND

REINDEER MEAT INSPECTION DECISION AND FEEDBACK

Official veterinarian	Date	Recipient	
Address	Reindeer herding cooperative		
Postal number and postal address	Reindeer abattoir, name		Abattoir number

MEAT INSPECTION DECISION AND REASONINGS

Meat inspection finding	Slaughter identification	Findings/inspected (n)	Condemned, whole carcass (n)	Partly condemned (n)	
				Parts of carcasses	Organs
**Ante mortem inspection (n)**		*	*		
Contamination (dirty fur)					
Eye infection					
Diarrhea					
Central nervous symptoms					
Wet belly					
Other reason					
Body condition class (1-4) of calves					
Abnormal fur					
Abnormal antler development (calf)					
**Post mortem inspection (n)**		*	*	*	*
Abnormal odor, color or structure					
Bruices					
Fractures					
Cahexia					
Tumor					
Aspiration					
Contamination					
Besnoitia					
Echinococcosis					
Deer ked (*Lipoptena cervi*)					
Lung changes					
Taenia hydatigena (cysticercus tenuicollis)					
Muscle cyst worm (*Taenia krabbei*)					
Warble fly (*Hypoderma tarandi*)					
Nose bot (*Cephenemyia trompe*)					
Setaria tundra					
Onchocerca					
Hot ear (*Lappnema auris*)					
Liver scars (parasitic granuloma)					
Other parasite					
Generalized inflammation					
Skin inflammation					
Abomasitis					
Pneumonia					
Pleuritis					
Pericarditis					
Arthritis					
Enteritis					
Stomatitis					
Papillomatosis					
Abscess					
Peritonitis					
Other inflammation					
Other reason					

* mandatory information
